# Macrophage source influences peritoneal persistence and immune cell dynamics in assays of reverse cholesterol transport

**DOI:** 10.1016/j.jlr.2026.101148

**Published:** 2026-09-15

**Authors:** Jordan M. Bobek, Cade R. Rahlf, Vera L. Tarakanova, Daisy Sahoo

**Affiliations:** 1 Department of Biochemistry, Medical College of Wisconsin, Milwaukee, WI, USA; 2 Department of Microbiology and Immunology, Medical College of Wisconsin, Milwaukee, WI, USA; 3 Cancer Center, Medical College of Wisconsin, Milwaukee, WI, USA; 4 Department of Medicine, Medical College of Wisconsin, Milwaukee, WI, USA; 5 Cardiovascular Research Center, Medical College of Wisconsin, Milwaukee, WI, USA

**Keywords:** cholesterol/efflux, transport, cholesterol/trafficking, inflammation, cytokines, lipoproteins/metabolism, BMDMs, J774 macrophages, innate immune activation

## Abstract

Assays to measure macrophage-to-feces reverse cholesterol transport (RCT) in mouse models are well-established, yet the immunologic consequences of injecting allogeneic versus syngeneic macrophages remain undefined in the context of this assay. In this study, we compared primary bone marrow-derived macrophages (BMDMs) and reticulosarcoma-derived J774 macrophages as cholesterol-loaded donor cells in a well-established in vivo RCT assay to determine whether macrophage origin influences host immune activation and cholesterol flux. Following intraperitoneal injection, J774 macrophages exhibited markedly reduced peritoneal persistence relative to BMDMs, with <1% of cells detectable at 24 or 48 h postinjection, compared to sustained retention of the BMDMs. This was accompanied by distinct immune activation profiles where J774 macrophages induced prolonged innate immune activation, whereas BMDMs elicited coordinated and transient responses involving early B cell activation that resolved by 48 h. Despite these differences in cellular persistence and immune response, RCT outcomes were comparable between groups under the conditions tested, with no major differences observed in hepatic uptake or fecal excretion of [^3^H]-cholesterol in BMDM-injected mice at 24 h compared to J774-injected mice. Mechanistically, these findings suggest that the presence of macrophages to mediate cholesterol efflux in the early steps of RCT likely dominates total RCT measurements, potentially buffering the impact of later differences in macrophage persistence and immune activation. Together, these results demonstrate that macrophage source influences peritoneal persistence and immune cell dynamics, but does not substantially alter RCT outcomes under the conditions tested, underscoring the importance of considering macrophage source when designing and interpreting macrophage-to-feces RCT studies.

HDL has well-established cardioprotective properties, primarily due to its critical role in facilitating reverse cholesterol transport (RCT) (1). In this process, HDL serves as the acceptor of excess cholesterol from cells within arterial plaque and delivers this peripheral cholesterol to the liver for excretion from the body via the feces (2). In 2003, Zhang *et al.* developed a novel assay to trace the transport of labeled cholesterol from macrophages to the feces in vivo, which relies on the injection of radiolabeled and cholesterol-loaded macrophages into the peritoneal cavity of mice. The movement of labeled cholesterol by endogenous mouse HDL into the blood, liver, and feces is then measured over the course of 48 h. This established assay has been widely adopted to evaluate how diverse biological variables influence macrophage-to-feces RCT in vivo. For example, how macrophage-to-feces RCT is impacted by HDL composition and function (3, 4, 5, 6), as well as cholesterol transporters and transfer enzymes (7, 8, 9, 10) has been widely studied. Furthermore, HDL’s ability to mediate RCT under conditions such as obesity (11), exercise (12), and inflammation (13, 14, 15) demonstrates the broad applicability of this assay in various physiological and pathological states.

The original RCT assay describes the use of cultured J774 macrophages, a murine macrophage-like cell line derived from a BALB/c mouse reticulum cell sarcoma and possessing a highly abnormal karyotype (16, 17). Since its original description, a wide range in macrophages from varying sources have been used in this assay including RAW 264.7 (3) and P388D1 cell lines (18). As in vivo studies are commonly performed in C57BL6/J mice, several groups have shifted to the use of primary peritoneal (14) and bone marrow-derived macrophages (BMDMs) (9) to avoid potential immunological implications with the macrophage-like cell lines from various backgrounds. While all sources of macrophages are still widely used and accepted in the field, most studies in the literature report the use of J774 macrophages.

It is well established that injection of allogenic cells elicits immune recognition through major histocompatibility complex (MHC) mismatch, leading to the activation of innate and adaptive immune pathways (19, 20, 21). The peritoneal cavity is immunologically active and enriched with resident macrophages and B-1 B cells (22, 23) that are capable of triggering rapid inflammatory responses to foreign stimuli (24, 25). Activation of these pathways can therefore contribute to early clearance of mismatched cells, such as J774 macrophages during the in vivo RCT assay (26). Consistent with this, De Beer *et al.* demonstrated that injection of allogenic J774 macrophages, but not syngeneic BMDMs, induced transient acute phase inflammation as measured by an elevation in serum amyloid A in plasma (13). Given that systemic inflammation has been shown to impair macrophage-to-feces RCT (15), these observations raise the possibility that immune activation following injection of allogeneic macrophages may confound interpretation of RCT outcomes.

Despite widespread use of the RCT assay, the immunologic consequences of injecting allogeneic macrophages into mice have not been rigorously examined in the context of this assay. Specifically, whether macrophage source alters peritoneal persistence, host immune activation and subsequent cholesterol flux during the in vivo RCT assay remains unclear. In this study, we tested the hypothesis that intraperitoneal injection of J774 macrophages into C57BL6/J mice, as compared to BMDMs, elicit host immune activation and alter peritoneal persistence within the peritoneal cavity. We demonstrate that as compared to BMDMs, J774 macrophages exhibit reduced residency in the peritoneal cavity, as reflected by decreased cell recovery at 24 and 48 h postinjection, and elicit distinct patterns of innate immune activation. Interestingly, despite the immunological differences, there were no differences observed in fecal cholesterol excretion at the endpoint of in vivo RCT with either macrophage type at 24 h. Together, this work demonstrates that, under the controlled conditions examined, RCT outcomes were resilient to differences in macrophage source, despite substantial differences in donor-cell peritoneal persistence and host immune activation, underscoring the importance of considering macrophage source when designing and interpreting macrophage-to-feces RCT studies.

## Materials and methods

### Materials

Ammonium-Chloride-Potassium lysis buffer, DNase I, high-capacity cDNA reverse transcriptase kit, SYBRGreen Master Mix, RPMI 1640, DMEM, Ecolite scinitallation fluid, beta actin (1:1000) antibody, and acetylated low density lipoprotein (acLDL) were purchased from Thermo Fisher Scientific (Waltham, MA). [1,2-3H(N)]-Cholesterol was purchased from Revvity (Waltham, MA). The Total RNA Purification Kit was purchased from Norgen Biotek Corp (Thorold, ON, Canada). ABCA1 antibody (1:1,000) was purchased from Abcam (Waltham, MA) and HRP-conjugated donkey-anti-rabbit-IgG (1:10,000) and sheep-anti-mouse IgG secondary (1:10,000) antibodies were purchased from Cytiva (Amersham). Recombinant mouse interferon-gamma was purchased from Biolgend (San Diego, CA). All other reagents used were of analytical grade.

### Animal studies

All studies were approved by the Institutional Animal Care and Use Committee of the Medical College of Wisconsin (AUA3282 and 971). C57BL/6J wild type mice and B6.SJL-*Ptprc*^*a*^ *Pepc*^*b*^/BoyJ mice (27) that encode the CD45.1 allele were acquired from The Jackson Laboratory (strain #000664 and #2014, Bar Harbor, ME) and were housed under normal light-dark cycle and bred in a pathogen-free barrier facility in accordance with federal and institutional guidelines. Male and female wild-type mice were used for bone marrow isolation and the in vivo studies between 8 and 12 weeks of age.

### Culturing and preparation of macrophage cell lines

Bone marrow was harvested from the femurs and tibias of wild-type C57BL/6J or B6.SJL-*Ptprc*^*a*^ *Pepc*^*b*^/BoyJ mice and cultured for 7 days in macrophage differentiation media (RPMI, 10% FBS, 1% penicillin-streptomycin, and 20% L929-conditioned media (source of macrophage colony stimulating factor). J774 macrophages, purchased from ATCC (Manassas, VA), were maintained in complete media (DMEM, 10% FBS, 200 mM glutamine, 1% penicillin/streptomycin) and used within 25 passages after thawing. For macrophage preparation for peritoneal exudate cell (PEC) analysis, bone marrow-derived or J774 macrophages were seeded at 3 × 10^6^ cells/T-75 flask and incubated in media with or without acLDL (25 μg/ml) for 24 h. Cells were washed and detached using EDTA-based dissociation. Briefly, cells were incubated with 1 mM EDTA in PBS and J774 macrophages were gently detached using a sterile Corning cell scraper, while BMDMs were detached by mechanical agitation with a syringe fitted with an 18-gauge needle. Following detachment, cells were centrifuged, resuspended in sterile PBS, and trypan blue-negative cells were counted using a hemocytometer. Cell suspensions were adjusted based on viable cell counts to a final concentration of 2 × 10^6^ cells/ml prior to aliquoting for intraperitoneal injection. To prepare macrophages for in vivo RCT studies, bone marrow-derived or J774 macrophages were seeded at 3 × 10^6^ cells/T-75 flask and incubated in labeling media containing acLDL (25 μg/ml) and [^3^H]-cholesterol (5 μCi/ml) for 24 h. Following washing, cells were equilibrated in serum-free media supplemented with 0.2% BSA for 4 h, and harvested as described previously prior to intraperitoneal injection.

### In vivo macrophage-to-feces RCT studies

*In vivo* RCT studies were performed as previously described with minor modifications (6). Briefly, C57BL/6J mice were housed in individual disposable cages with minimal bedding for 24 h before intraperitoneal injection of radiolabeled macrophages (∼1 × 10^6^ DPM/1 × 10^6^ trypan blue negative cells per mouse). Blood was collected via submental bleeds prior to macrophage injection and at 4 and 24 h thereafter. Mice were euthanized at 24 h postinjection, and the livers and total feces excreted during the 24-h experiment were harvested for analysis. Plasma was separated from blood samples via centrifugation and 20 μl was mixed with 4 ml of scintillation fluid prior to counting on a liquid scintillation counter. Liver (∼50 mg) was weighed and lipids extracted using the Bligh and Dyer method (28). Total fecal samples were weighed, smashed under liquid nitrogen, and lipids were extracted using a modified Folch method (29). Liver and fecal neutral sterol lipids were dried down and resuspended in toluene prior to counting of radioactivity. Plasma, liver, and fecal [^3^H]-cholesterol counts are expressed as a percentage of total [^3^H]-cholesterol injected.

### PEC isolation

C57BL/6J mice were injected intraperitoneally with peritoneal macrophages or BMDMs (∼1 × 10^6/^ cells/mouse) and euthanized 24 or 48 h postinjection. PECs were harvested by peritoneal lavage and centrifugation. Contaminating red blood cells were lysed with ammonium-chloride potassium lysis buffer, cells were pelleted by centrifugation and resuspended in complete media for counting.

### Flow cytometry

Single cell suspensions of the isolated PECs were prepared in fluorescence-activated cell sorting buffer (phosphate-buffered saline, 2% FBS) and incubated with fluorescently-labeled antibodies. Data were acquired using the Celesta flow cytometer (BD Biosciences, Franklin Lakes, NJ), and data were analyzed using FlowJo software (BD Biosciences, Franklin Lakes, NJ). The following antibodies used in this study were purchased from Biolegend (San Diego, CA): CD45R/B220-PEDazzle 594 (cat. 103258), CD19-FITC (cat. 115506), CD80-APC (cat. 104714), CD86-PECy7 (cat. 105013), CD69-PB (cat. 104523), CD3-PB (cat. 100214), CD4-PeCy5 (cat. 100410), CD8a-FITC (cat. 100706), CD44-APC (cat. 103012), CD62L-PEDazzle594 (cat. 104448), F4/80-APCCy7 (cat. 123118), CD45.1-PE (cat. 110707), I-A/I-E (cat. B361458), and H-2Dd-FITC (cat. 110606).

### RNA isolation and quantitative reverse transcriptase PCR

Isolated PECs were washed with PBS and total RNA was extracted using the Total RNA Purification Kit following the manufacturer’s instructions. Contaminating DNA was digested with DNase I before 1 μg total RNA was reverse-transcribed into single-stranded cDNA using the high-capacity cDNA reverse transcriptase kit. Quantitative RT-PCR was performed with SYBRGreen Master on a CFX Opus qPCR Instrument (BioRad). Relative gene expression was determined using the 2^-ΔΔCt^ method with *Actb* serving as the internal control. Primers were designed and purchased from IDT Technologies (Coralville, IA). Primer sequences are detailed in Supplemental Table S1.

### Immunoblot analysis

Cell lysates (25 μg protein) were mixed with an equal volume of Laemmli sample buffer containing 5% β-mercaptoethanol and heated at 95°C for 5 min. Proteins were separated on 8% SDS-polyacrylamide gels and transferred to nitrocellulose membranes by wet transfer. Membranes were blocked with 5% nonfat dry milk prepared in Tris-buffered saline containing 0.1% Tween-20 for 1 h at room temperature and incubated overnight at 4°C with primary antibodies diluted in 1% milk. Membranes were washed three times with Tris-buffered saline containing 0.1% Tween-20 and then incubated with HRP-conjugated secondary antibodies diluted in 1% milk for 1 h at room temperature. Protein bands were detected using SuperSignal™ West Pico or SuperSignal™ West Femto chemiluminescent substrate (Thermo Fisher Scientific) and imaged with a ChemiDoc™ MP Imaging System (Bio-Rad). Band intensities were quantified using Image Lab software (Bio-Rad) and normalized to β-actin.

### Data and statistical analysis

For animal studies, each animal represents a biological replicate, where every experiment had at least 6 or more mice per group per experiment. For flow cytometry experiments, frequencies and medium fluorescence values (MFI) were determined using FlowJo v10.0 Software (BD Biosciences, Franklin Lakes, NJ). For whole animal and macrophage isolation studies, male and female data were analyzed separately for sex-specific differences. As no significant sex-specific differences were observed, data presented in this study are pooled from both sexes. Statistical analyses were performed using GraphPad Prism (San Diego, CA), with details of each analysis provided in Figure Legends.

## Results

### J774 macrophages exhibit reduced peritoneal persistence compared to BMDMs

It is important that macrophages injected into the peritoneal cavity remain in the peritoneum long enough to participate in HDL-mediated cholesterol efflux during the early steps of the in vivo RCT assay. We therefore compared the persistence of J774 macrophages and BMDMs isolated from B6.SJL-*Ptprc*^*a*^ *Pepc*^*b*^/BoyJ mice, with or without cholesterol loading, in the peritoneal cavity 24 and 48 h after injection. B6.SJL-*Ptprc*^*a*^ *Pepc*^*b*^/BoyJ BMDMs were used to enable quantification of donor and recipient macrophages by detection of CD45.1 (donor) versus CD45.2 (recipient) alleles by flow cytometry. J774 macrophages were identified based on expression of H2-D^d^, which distinguishes them from C57BL/6 recipient macrophages that are H2-D^d^-negative (Supplemental Fig. S1). For simplicity, cholesterol-loaded (CL) J774 macrophages or BMDMs will be referred to as “CL-J774s” or “CL-BMDMs” throughout the article.

In J774-injected mice at 24 h postinjection, 0.14% of PECs were H2-D^d+^, compared to 2.68% CD45.1^+^ PECs in BMDM-injected mice (Supplemental Fig. S1B, C). By 48 h, 0.08% of PECs were H2-D^d+^ in J774-injected mice versus 1.20% CD45.1^+^ in BMDM-injected mice (Supplemental Fig. S1B, C). The percentage of macrophages remaining in the peritoneum was calculated as the absolute number of H2-D^d+^ or CD45.1^+^ cells recovered (Fig. 1A, B), derived from total peritoneal lavage cell counts (Supplemental Fig. S2D), divided by the 1 × 10^6^ cells initially injected into each mouse. These data further highlight that less than 1% of the injected J774 macrophages are recovered in the peritoneal cavity after 24 and 48 h while ∼8% and 5% of injected BMDMs are recovered at 24 and 48 h, respectively (Fig. 1C). Cholesterol loading did not have a statistically significant effect on macrophage persistence with either source.

**Fig. 1:**
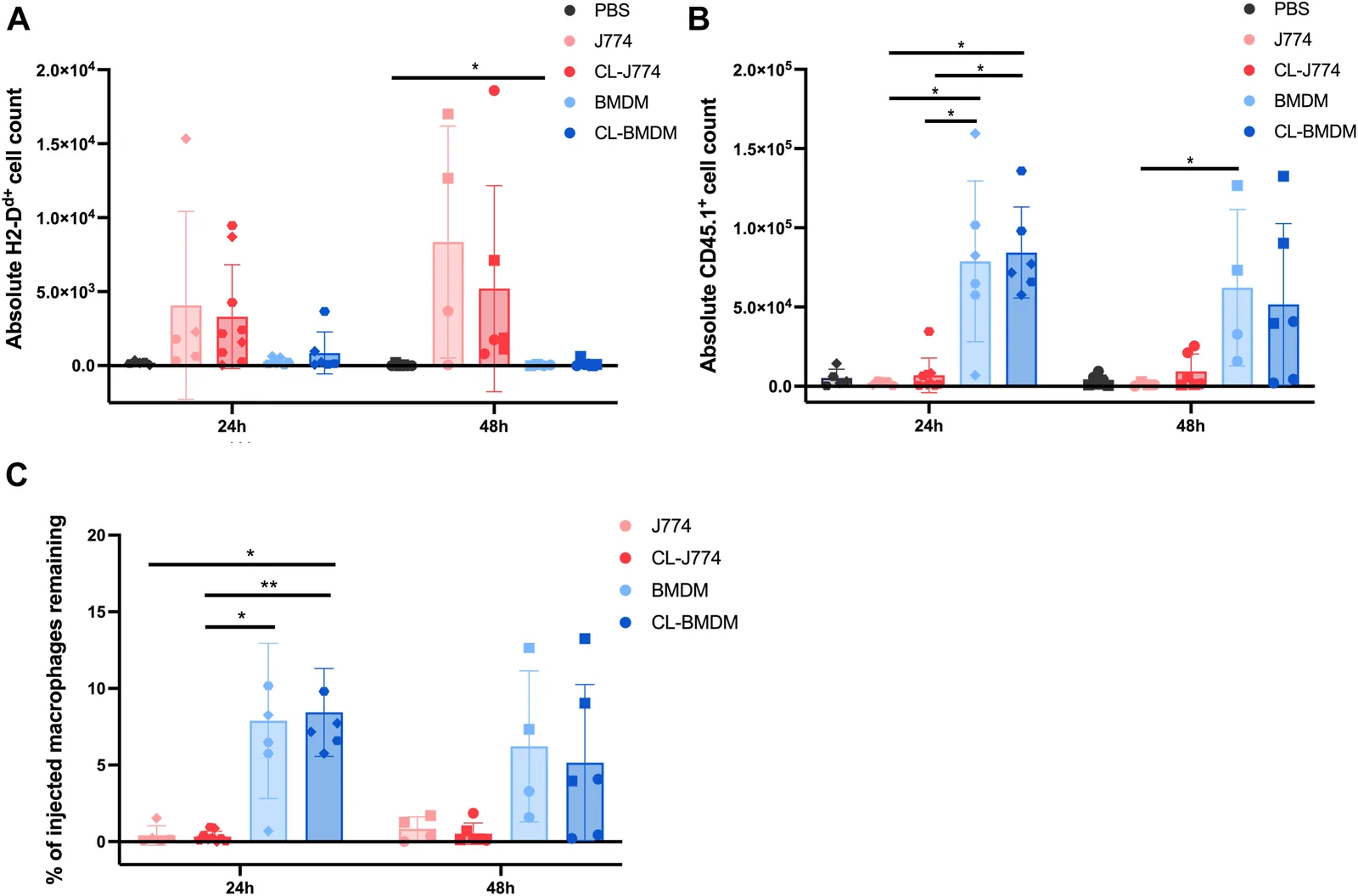
J774 macrophages exhibit reduced peritoneal persistence compared to BMDMs. C57BL/6J wild type mice (n = 4–9) were injected with PBS, J774 macrophages, or BMDMs (1 × 10^6^ cells/mouse) that were preincubated with or without 25 μg/ml acLDL for 24 h to load cells with cholesterol (“CL-J774” or “CL-BMDM”). Peritoneal exudate cells (PECs) here harvested by peritoneal lavage 24 or 48 h postinjection and cell suspensions were prepared independently for flow cytometry analysis. Absolute number of (A) H2-D^d+^ and (B) CD45.1^+^ cells were determined using the frequencies of these populations. C:) The percentage of the remaining injected J774 macrophages and BMDMs were calculated using corresponding absolute numbers. Statistical analyses were performed separately for the 24 h and 48 h datasets using Kruskal-Wallis tests followed by Dunn’s multiple comparisons test for all pairwise comparisons within each time point. ∗*P* < 0.05, ∗∗*P* < 0.01. acLDL, acetylated low density lipoprotein; BMDM, bone marrow-derived macrophage.

### J774 macrophage injection induces sustained peritoneal macrophage activation

To understand potential mechanisms underlying the differences in macrophage persistence, we examined host immune responses following injection of J774 macrophages and BMDMs. To determine whether macrophage origin alters early peritoneal immune activation, PECs were harvested at 24 and 48 h postintraperitoneal injection of J774 macrophages or BMDMs with or without cholesterol loading to mimic conditions during in vivo macrophage-to-feces RCT. Select cell surface markers commonly associated with activated macrophages and B-1 B cells of the recipient were measured using flow cytometry.

Macrophage activation markers were analyzed on cells pregated as F4/80^+^ and CD45.1^-^ host peritoneal macrophages (Supplemental Fig. S2A) to assess the innate immune response. Macrophage activation was assessed by expression of MHC-II and CD80, the latter being a costimulatory protein that plays an important role in T cell priming and stimulation. At 24 h postinjection, the frequency of CD80^+^ and MHCII^hi^ PEC macrophages increased across all groups compared to control mice injected with PBS (Fig. 2A, B). The mice injected with J774 or CL-J774 macrophages had an average of 17.7% and 23.4% increases in CD80^hi^ macrophages and 19.7% and 15.2% increases in MHCII^hi^ macrophages as compared to PBS-injected mice (Fig. 2A, representative histograms shown below the pooled data). The mice injected with BMDMs or CL-BMDMs had an average of 17.3% and 22.5% increases in CD80^hi^ macrophages and a 17.5% increase in MHCII^hi^ macrophages in BMDM-injected mice only as compared to PBS-injected mice (Fig. 2B, representative histograms shown below the pooled data). By 48 h postinjection, CD80^+^ and MHCII^hi^ PEC macrophage frequencies in mice injected with J774 and CL-J774 macrophages remained above PBS control levels, however these differences did not reach statistical significance. In contrast, frequencies in BMDM- and CL-BMDM-injected mice appeared to return toward baseline.

**Fig. 2:**
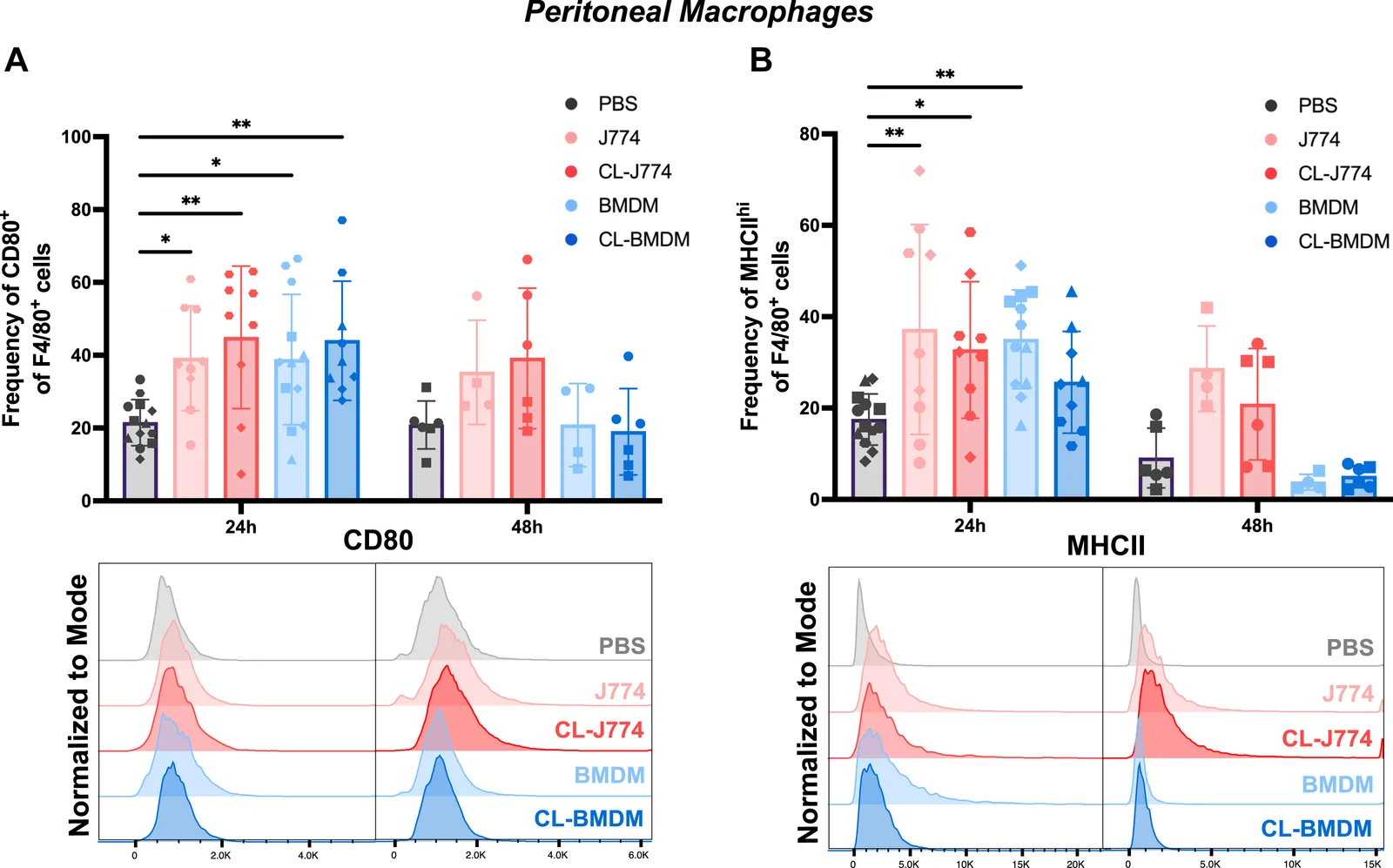
J774 macrophage injection induces sustained peritoneal macrophage activation. Wild type mice (n = 9–13) were injected with PBS, J774 macrophages, or BMDMs (1 × 10^6^ cells/mouse) that were preincubated with or without 25 μg/ml acLDL for 24 h (“CL-J774” or “CL-BMDM”). Peritoneal exudate cells (PECs) here harvested by peritoneal lavage 24 or 48 h postinjection and cell suspensions were prepared independently for flow cytometry analysis. Frequencies and MFIs of (A) CD80^+^ or (B) MHCII^hi^ cells on F4/80^+^ gated cells were determined and compared between groups. Gating was performed such that PBS-injected control groups exhibited comparable baseline frequencies across independent experiments. Statistical analyses were performed separately for the 24 h and 48 h datasets using Kruskal-Wallis tests followed by Dunn’s multiple comparisons test comparing each treatment group with the PBS control within each time point. ∗*P* < 0.05, ∗∗*P* < 0.01. acLDL, acetylated low density lipoprotein; BMDM, bone marrow-derived macrophage; MFI, mean fluorescence intensity.

### J774 macrophage injection drives selective B-2 B cell activation at 48 h postinjection

B-2 B cells develop in the bone marrow with subsequent localization and differentiation in the secondary lymphoid organs throughout the life of the host. In contrast, B-1 B cells arise early in embryogenesis, reside in the body cavities, including peritoneal cavity, have a limited diversity of B cell receptors, and are maintained by self-renewal (30). Interestingly, B-2 B cells present in the peritoneal cavity acquire phenotype that is intermediate between B-1 B cells and splenic B-2 B cells, suggesting that they are not transiently circulating through the body cavities (31). B cell activation was evaluated as they serve as antigen-presenting cells capable of promoting T cell activation through expression of costimulatory molecules, and we hypothesized that differential B cell activation might contribute to differences in macrophage persistence following intraperitoneal injection. B cells were analyzed as B-1 (CD19^+^B220^lo^) and B-2 (CD19^+^B220^hi^) populations to assess their activation status (Supplemental Fig. S2B), measured by expression of CD86, another costimulatory protein, and CD69, an early B cell activation marker. At 24 h postinjection, only the BMDM and CL-BMDM injected mice showed increases in the frequency of CD69^+^ and CD86^+^ PEC B-1 B cells as compared to PBS control mice (Fig. 3A, B, representative histograms shown below pooled data). PEC B-1 B cells from BMDM-injected mice averaged a 19.2% increase in CD69^hi^ B1 cells and an average of 20% increase in CD86^hi^ B1 cells^,^ while, on average, the CL-BMDM injected mice had a 20.7% increase in CD69^hi^ B1 cells as compared to PBS-injected mice. By 48 h postinjection, there were no statistical differences in CD69 or CD86 expression across groups, however the frequency of CD69^+^ PEC B-1 B cells remained elevated in the BMDM and CL-BMDM injected mice. In contrast, injection of J774 macrophages did not alter B-1 B cell activation at either time point, regardless of cholesterol status.

**Fig. 3:**
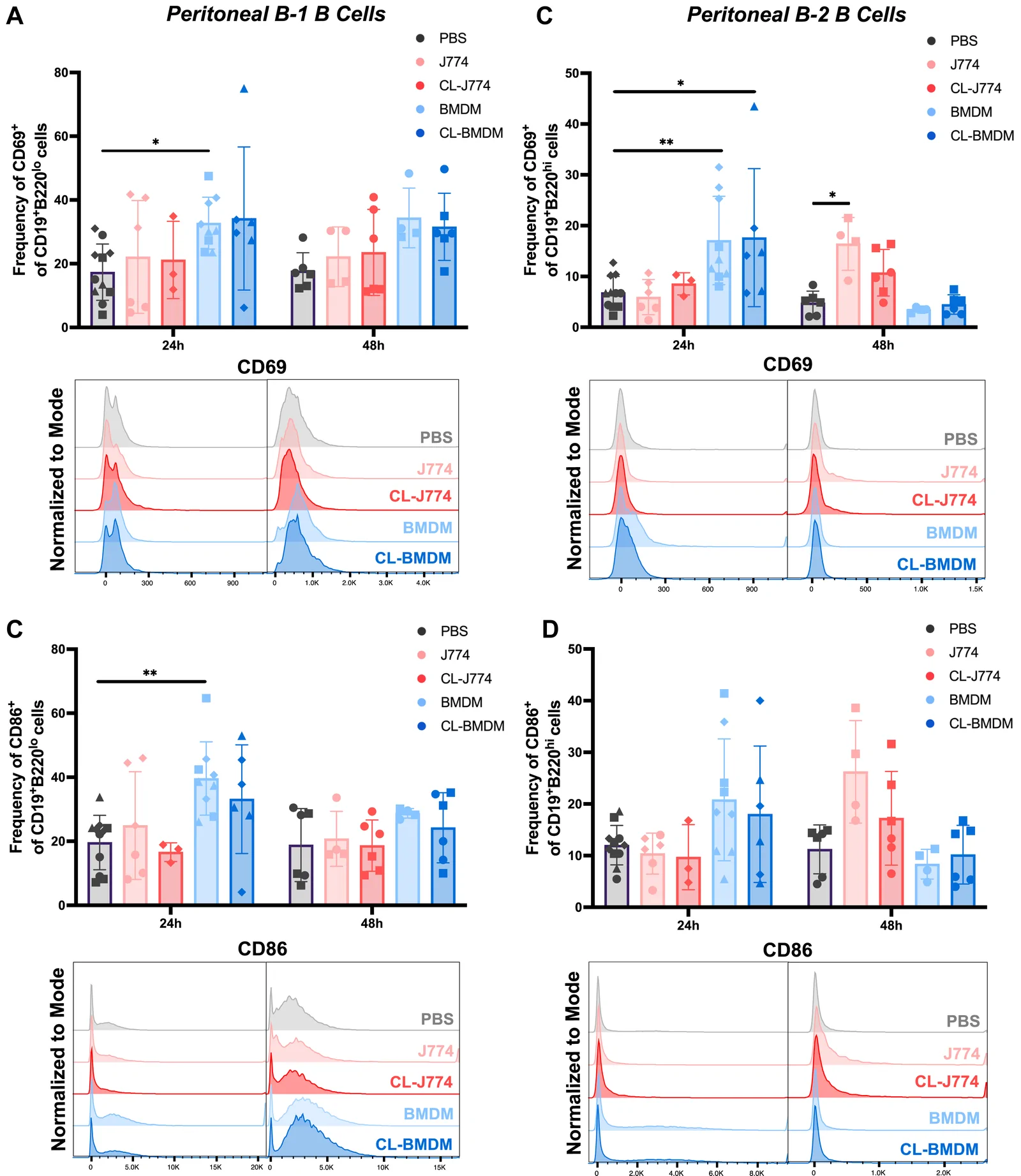
J774 macrophages injection drives selective B-2 B cell activation at 48 h postinjection. Wild type mice (n = 9–13) were injected with PBS, J774 macrophages, or BMDMs (1 × 10^6^ cells/mouse) that were preincubated with or without 25 μg/ml acLDL for 24 h (“CL-J774” or “CL-BMDM”). Peritoneal exudate cells (PECs) were harvested by peritoneal lavage 24 or 48 h postinjection and cell suspensions were prepared independently for flow cytometry analysis. Frequencies and MFIs of (A) CD69^+^ or (B) CD86^+^ cells gated on CD19^+^B220^lo^ cells were determined and compared between groups. Frequencies and MFIs of (C) CD69^+^ or (D) CD86^+^ cells gated on CD19^+^B220^hi^ cells were determined and compared between groups. Gating was performed such that PBS-injected control groups exhibited comparable baseline frequencies across independent experiments. Statistical analyses were performed separately for the 24 h and 48 h datasets using Kruskal-Wallis tests followed by Dunn’s multiple comparisons test comparing each treatment group with the PBS control within each time point. ∗*P* < 0.05, ∗∗*P* < 0.01. acLDL, acetylated low density lipoprotein; BMDM, bone marrow-derived macrophage; MFI, mean fluorescence intensity.

At 24 h postinjection, BMDM and CL-BMDM groups exhibited increased frequencies of CD69^+^ and CD86^+^ PEC B-2 B cells compared to PBS control mice (Fig. 3C, D, representative histograms shown below pooled data). Only the increase in CD69^+^ was statistically significant (10.3% and 10.8% increases, respectively). By 48 h postinjection, these frequencies returned to baseline in the BMDM- and CL-BMDM-injected groups. In contrast, increased CD69^+^ and CD86^+^ PEC B-2 B cell frequencies were observed in J774-injected mice as compared to PBS control mice, while CL-J774 injected mice exhibited a similar trend. However, only statistically significant increases were only observed in J774 macrophage-injected mice, where CD69^+^ increased by 11.6% and CD86^+^ increased by 15% as compared to PBS controls.

To further assess whether differences in macrophage persistence were associated with altered immune cell recruitment, host peritoneal immune cell populations were quantified following exclusion of donor macrophages from F4/80^+^ gate based on H2D-d or CD45.1 expression. No evidence of increased host macrophage or T-cell recruitment was observed following J774 macrophage injection, although BMDM-injected mice exhibited a modest increase in B-1 B cells at 48 h postinjection (Supplementary Fig. S2C, D). T cell activation (CD44/CD62L) did not differ between groups (data not shown).

### CL-J774 macrophages induce a proinflammatory interferon-γ response in PECs

To determine whether these early immune differences were accompanied by changes in T cell-associated and inflammatory gene expression within the peritoneal environment, PECs harvested from C57BL/6 mice 24 h postinjection of CL-J774 macrophages or CL-BMDMs were analyzed by qPCR for expression of the T cell marker gene *Cd3e* (CD3ε) and the proinflammatory cytokine *Ifng* (interferon-γ). PECs from mice injected with CL-J774 macrophages exhibited a 1.72-fold increase of *Ifng* expression compared to CL-BMDM injected mice (Fig. 4A). Interestingly, this increase was not accompanied by differences in T cell marker *Cd3e* expression (Fig. 4B). A previous study reported that 48-h exposure to interferon-γ exposure decreased macrophage ABCA1 expression, which correlated with reduced [^3^H]-cholesterol efflux capacity (32). To determine whether shorter exposure times relevant to our experimental model produced similar effects, J774 macrophages were treated with mouse interferon-γ for 6 and 24 h. Interferon-γ treatment did not alter ABCA1 protein expression at either time point, even at supraphysiological concentrations of 100 U/ml (Fig. 4C, D).

**Fig. 4:**
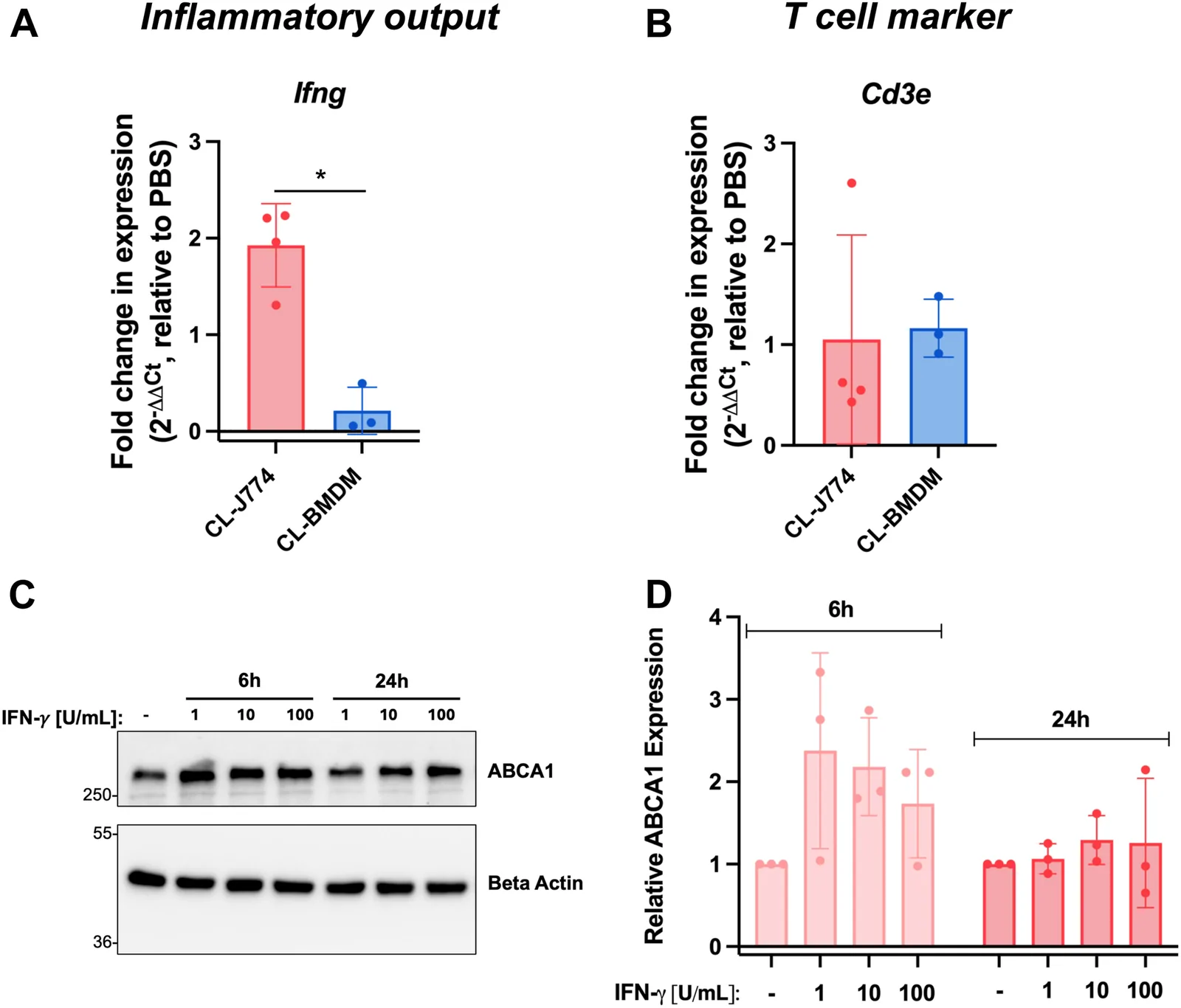
J774 macrophages increase peritoneal exudate cell (PEC) interferon-γ expression. Wild type mice were injected with PBS (n = 2), J774 macrophages (n = 8), or BMDMs (n = 6) (1 × 10^6^ cells/mouse) that were preincubated with 25 μg/ml acLDL for 24 h (“CL-J774” or “CL-BMDM”). PECs were harvested by peritoneal lavage 24 h postinjection and samples from two mice per group were pooled prior to RNA isolation. RNA was reverse transcribed into cDNA and analyzed by qPCR using primers specific for (A) *Ifng* and (B) *Cd3e*. Relative gene expression was normalized to *Actb* and calculated relative to PBS treatment using the 2^-ΔΔCt^ method. Statistical analyses was performed on ΔCt values using unpaired two-tailed Student’s t-tests. ∗*P* < 0.05. C: J774 macrophages were treated with 1, 10, and 100 U/ml of mouse interferon-γ for 6 and 24 h. ABCA1 expression was assessed by immunoblot analyses. D: Densitometry was performed by normalizing expression to housekeeping gene, beta-actin. Statistical analyses were performed using Kruskal-Wallis tests followed by Dunn’s multiple comparisons test comparing all groups. acLDL, acetylated low density lipoprotein; BMDM, bone marrow-derived macrophage; qPCR, quantitative polymerase chain reaction.

### Macrophage source does not impact in vivo macrophage-to-feces RCToutcomes

To investigate whether macrophage source influences in vivo RCT, the movement of [^3^H]-cholesterol from intraperitoneal J774 macrophages or BMDMs into the plasma, liver, and feces was measured over a 24-h period. In both groups, [^3^H]-cholesterol counts in the plasma accumulated over the 24 h, reaching approximately 1.5% of the injected dose (Fig. 5A). The BMDM-injected mice had a modest, but not statistically significant 0.38% increase in plasma radioactivity as compared to the J774 injected mice at this 24-h time point. In contrast, the amount of [^3^H]-cholesterol in the liver after 24 h was comparable between groups, with 3.45% of the injected dose detected in J774-injected mice and 4.24% in BMDM-injected mice (Fig. 5B). Similarly, fecal excretion of [^3^H]-cholesterol was low and not markedly different between conditions, measuring 0.24% and 0.35% of the injected dose in J774-and BMDM-injected mice, respectively (Fig. 5C).

**Fig. 5:**
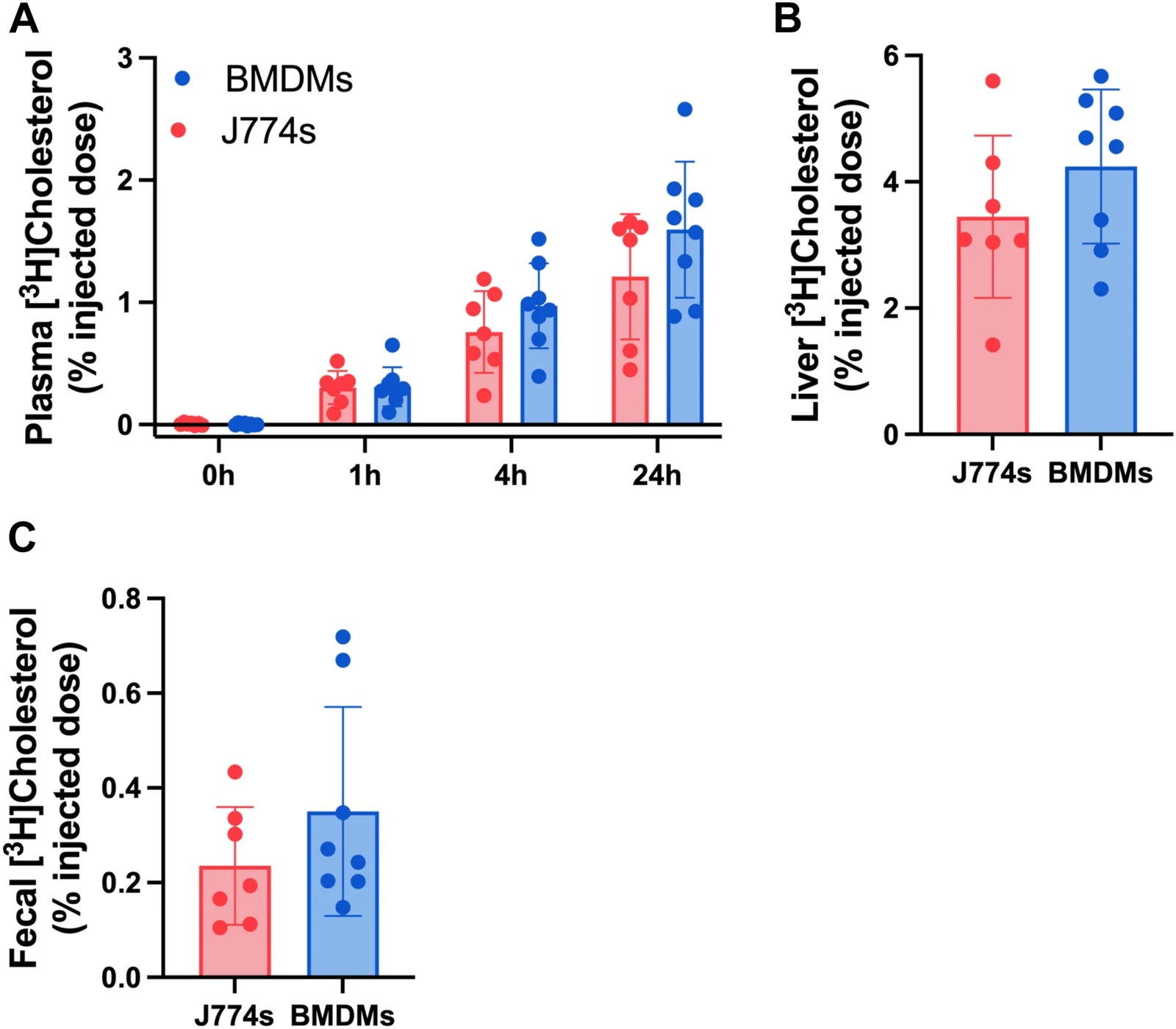
Macrophage source does not significantly change in vivo macrophage-to-feces reverse cholesterol transport outcomes. Wild type mice (n = 7–10) were intraperitoneally injected with [^3^H]-cholesterol-labeled and acLDL-loaded J774 macrophages or BMDMs (1 × 10^6^ cells/mouse). A: Plasma radioactivity (DPM) was monitored over the course of 24 h and radioactivity associated with liver (B) and fecal (C) lipids were measured after the mice were euthanized at 24 h postinjection. Values are represented as percentage of injected macrophage dose. For plasma, liver, and feces data, statistical significance was determined using a two-tailed Mann-Whitney U test. Plasma data were analyzed separately at each time point. acLDL, acetylated low density lipoprotein; BMDM, bone marrow-derived macrophage.

## Discussion

This study demonstrates that while macrophage source significantly influences peritoneal persistence and immune activation, it does not substantially alter in vivo macrophage-to-feces RCT under the conditions examined here. Specifically, when J774 macrophages are intraperitoneally injected, they exhibited markedly reduced persistence and induced a distinct pattern of immune activation compared to BMDMs. Although these differences did not alter the RCT endpoint in the present study, the associated immune activation may influence the interpretation of in vivo HDL-mediated RCT measurements, particularly in the early phases of the assay, and potentially in experimental systems involving genetic or pharmacologic perturbations. Moreover, the differences in persistence and immune activation suggest that primary macrophages may be preferable for experimental questions in which prolonged donor-cell residence or minimization of donor-cell-associated immune activation is important.

Effective macrophage-to-feces RCT requires that injected macrophages persist in the peritoneal cavity long enough to participate in cholesterol efflux with endogenous mouse HDL. However, we observed striking differences in macrophage retention, with fewer than 1% of injected J774 macrophages remaining in the peritoneal cavity at 24 and 48 h postintraperitoneal injection. In contrast, a substantial fraction of BMDMs persisted (∼8% at 24 h and ∼ 5% at 48 h). These differences suggest that J774 macrophages are rapidly recognized and eliminated by host immune mechanisms, whereas BMDMs are retained longer, allowing for sustained functional interactions within the peritoneal environment. Although analysis of host peritoneal immune cell populations did not demonstrate increased recruitment of macrophages or T cells following J774 injection (Supplemental Fig. S2C), additional studies directly assessing donor macrophage survival, apoptosis, and phagocytic clearance will be necessary to define the mechanisms responsible for their reduced persistence in the peritoneal cavity.

To investigate potential mechanisms underlying the differences in peritoneal persistence, we examined host immune activation and found distinct patterns of immune responses that were dependent on macrophage source. Injection of J774 macrophages induced sustained activation of resident macrophages with minimal early B cell activation, that was accompanied by an increase in B-2 B cell activation at 48 h. In contrast, injection of BMDMs elicited a more coordinated and transient immune response, characterized by early activation of macrophages, B-1 and B-2 B cells that resolved by 48 h. Consistent with these immune phenotypes, *Ifng* expression was increased following CL-J774 macrophage injection. However, the absence of changes in *Cd3e* expression suggests that elevated interferon-γ signaling unlikely reflects increased T cell abundance. Instead, this may reflect heightened activation of existing T cell populations or contributions from innate lymphocytes within the peritoneal cavity. As interferon-γ has been reported to decrease ABCA1 expression and cholesterol efflux in macrophages (32), we hypothesized that increased interferon-γ production postinjection of J774 macrophages could contribute to early differences in the RCT process by reducing ABCA1 expression. Instead, we found that exposure of J774 macrophages to physiological (1 U/ml) or supraphysiological concentrations (100 U/ml) of interferon-γ did not decrease ABCA1 expression in J774 macrophages. These findings suggest that increased interferon-γ production at the levels observed following CL-J774 injection is unlikely to impair ABCA1-mediated HDL efflux during the in vivo RCT assay. Together with the peritoneal persistence data, these immunologic findings suggest that J774 macrophages are rapidly recognized and eliminated by the host immune system with corresponding increase in immune activation. Conversely, BMDMs had measurable persistence in the peritoneal cavity and injection was associated with transient increases in macrophages and B cell activation markers that were not sustained at later time points.

Despite these differences in peritoneal persistence and immune activation, 24-h RCT outcomes were largely unchanged between groups under the conditions tested. The only potential difference observed was modestly elevated plasma [^3^H]-cholesterol at 24 h postinjection of BMDMs as compared to J774 macrophages. This increase suggests enhanced efflux of [^3^H]-cholesterol from the BMDMs into HDL as compared to the J774 macrophages during the 24-h period. One explanation for the overall similarity in RCT measurements between groups is that cholesterol efflux occurs predominantly during the early postinjection period, which could be prior to the substantial J774 macrophage clearance or divergence in immune responses. Previous studies demonstrated rapid increases in plasma [^3^H]-cholesterol within the first 6 h following macrophage injection (10), indicating that early macrophage persistence is likely sufficient to account for the majority of measurable RCT. Thus, even transiently-retained J774 macrophages may contribute adequately to early efflux, resulting in comparable RCT outcomes.

An additional consideration is whether the relationship between macrophage source and RCT observed here is maintained in disease-relevant recipient models. While the original macrophage-to-feces RCT model was established in chow-fed C57BL/6J recipients (6), the assay is has also been performed in hyperlipidemic models, including ApoE-deficient mice and genetically modified LDLR-deficient backgrounds, as well as mice-fed Western or high-fat diets, (33, 34, 35, 36, 37). The use of chow-fed C57BL/6J recipients in the present study was intentional, as this controlled setting allowed us to examine the effects of donor macrophage source on host immune activation and donor-cell persistence without the additional influence of preexisting hyperlipidemia or systemic inflammation, as these conditions can independently exaggerate immune responses, alter lipoprotein metabolism and cholesterol trafficking, and may therefore influence the effects of macrophage source on RCT. Future studies to compare RCT using J774 macrophages versus BMDMs in disease-relevant models are warranted.

These findings also raise the possibility that exogenous macrophage-induced immune activation may represent an underappreciated variable in the interpretation of RCT assays. Inflammation has been shown to influence lipoprotein remodeling as well as hepatobiliary cholesterol trafficking processes that decrease in vivo RCT outcomes (15). It has also been demonstrated in vitro that inflammatory factors such as interferon-γ and proinflammatory cytokines can alter HDL composition or efflux functionality with macrophages (38, 39). Although not directly assessed in this study, the distinct immune responses elicited by J774 macrophages could influence these processes and thereby affect cholesterol flux independently of macrophage persistence. This highlights the importance of considering potential inflammatory effects on key biological processes involved in RCT when interpreting in vivo RCT data.

Our results are consistent with prior studies demonstrating intrinsic differences between J774 macrophage-like cells and primary BMDMs. Notably, intraperitoneal injection of J774 macrophages, but not BMDMs, into C57BL/6 mice has been shown to induce transient acute phase responses in vivo (13), highlighting distinct immunogenic properties between these cell types. These in vivo differences are supported by a broader body of literature describing fundamental disparities between macrophage-like cell lines, such as J774 macrophages, and primary BMDMs. Proteomic analyses have shown that J774 macrophages, while one of the closest macrophage-like cell lines to primary macrophages, remain substantially divergent in global protein expression and immune-related pathways as compared to BMDMs (40). Functional transcriptomic studies further demonstrate that J774 macrophages exhibit a delayed and attenuated response to bacterial infection, with reduced magnitude and altered composition of differentially expressed immune genes compared to BMDMs (41). J774 macrophages and BMDMs also exhibit intrinsic differences in the regulation of cholesterol efflux. In particular, cAMP is often used to induce ABCA1-mediated cholesterol efflux in in vitro studies of J774 macrophages (42), but not BMDMs, highlighting differences in efflux regulation between these cell types. In our study, J774 macrophages were evaluated without pharmacologic cAMP stimulation, consistent with prior macrophage-to-feces RCT studies using J774 macrophages (4, 7, 13, 15, 43, 44). As such, future studies should examine whether RCT outcomes are influenced by differences in regulation of efflux based on the type of macrophage used for these assays.

In addition, earlier work has shown that J774 macrophages have reduced cytokine production and inflammatory activation dynamics relative to primary macrophages, supporting the concept that these cells possess shifted immune activation thresholds and do not fully recapitulate physiological macrophage responses (45). Collectively, these studies reinforce that J774 macrophage-like cells and primary BMDMs differ substantially in both baseline state and stimulus-driven immune function, providing mechanistic basis for the differential immune activation and peritoneal persistence observed in our studies.

Several limitations of this study should be considered when interpreting these findings. First, the in vivo RCT assay provides a cumulative measure of cholesterol flux and does not give insight on the contribution of early versus late efflux events nor does it distinguish cell-specific sources of cholesterol movement. As a result, differences in macrophage behavior during the initial postinjection period may not be fully captured by the 24-h endpoint of our assays. This time point was selected because interpretation of RCT at later time points may have been complicated by the markedly reduced recovery of J774 macrophages from the peritoneal cavity at 48 h, thus making it difficult to distinguish effects of macrophage source from differences in macrophage persistence. Second, all experiments were performed in chow-fed C57BL/6J recipient mice, and therefore our findings cannot establish whether the effects of macrophage source on donor-cell persistence, immune activation, or RCT are maintained in hyperlipidemic or inflammatory recipient models. Third, the efficiency of peritoneal lavage recovery was not formally assessed immediately following intraperitoneal injection. However, all groups were subjected to the same standardized lavage procedure, and our conclusions are based on relative differences in recovered cells under standardized conditions. In addition, total PEC counts recovered from lavages were comparable in magnitude across experiments and treatment groups (Supplemental Fig. S2D), indicating that large differences in overall cell recovery were not observed. We also cannot exclude the possibility that differential cell adherence to peritoneal surfaces contributed to differences in macrophage recovery. Furthermore, as immune responses were assessed at 24 and 48 h postinjection only, it is possible that earlier or later time points could reveal other differences, as well as changes in T cell recruitment measured by qPCR that was not detected at 24 h postinjection. In addition, gene expression was assessed in bulk PECs, which precludes cell-type-specific attribution of the observed changes in *Ifng* expression to a defined immune cell population. Finally, while our data suggest that inflammatory signaling differs between injection of macrophage types, we did not directly assess downstream effects on HDL particle composition or cholesterol handling. These mechanisms remain important areas for future investigation to fully define how immune context may intersect with cholesterol transport pathways in vivo. In addition, future studies could further interrogate how macrophage viability influences RCT outcomes by comparing live versus non-viable labeled donor macrophages. Similarly, extending these comparisons to macrophages derived from BALB/c mice may help clarify whether the observed differences are dependent on donor strain.

In summary, macrophage origin is an important experimental variable that influences donor-cell persistence and host immune activation during the macrophage-to-feces RCT assay. Under the conditions examined in this study, J774 macrophages and BMDMs produced comparable in vivo RCT outcomes, despite substantial differences in donor-cell persistence and host immune responses. These findings indicate that the 24-h RCT endpoint can be resilient to differences in macrophage persistence and immune activation, while highlighting additional biological considerations that may be relevant when selecting macrophage sources and interpreting RCT studies. More broadly, our findings support careful consideration of macrophage source and experimental context when designing and interpreting macrophage-to-feces RCT studies.

## Data availability

The data underlying this study are available from the corresponding author upon reasonable request.

## Supplemental data

This article contains supplemental data.

## Conflict of interest

The authors declare that they have no conflicts of interest with the contents of this article.
