# Supplementary material for: Macrophage source influences peritoneal persistence and immune cell dynamics in assays of reverse cholesterol transport
Source: J Lipid Res. 2026 Sep 15;67(10):101148. doi: 10.1016/j.jlr.2026.101148 (PMC13675811; doi:10.1016/j.jlr.2026.101148)
Supplement: Supplementary file 1 [file mmc1.docx]

**SUPPLEMENTAL INFORMATION:**

**Macrophage source influences peritoneal persistence and immune cell dynamics in assays of reverse cholesterol transport**

Jordan M. Bobek^1^, Cade R. Rahlf^2^, Vera L. Tarakanova^2,3^, and Daisy Sahoo^1,3,4,5^

^1^Department of Biochemistry, Medical College of Wisconsin, Milwaukee, Wisconsin, USA

^2^Department of Microbiology and Immunology, Medical College of Wisconsin, Milwaukee, Wisconsin, USA

^3^Cancer Center, Medical College of Wisconsin, Milwaukee, Wisconsin, USA

^4^Department of Medicine, Medical College of Wisconsin, Milwaukee, Wisconsin, USA

^5^Cardiovascular Research Center, Medical College of Wisconsin, Milwaukee, Wisconsin, USA

**Supplemental Figure 1. PEC gating strategy for CD45.1^+^ or H-2Dd^+^ cells.**

Wild type mice (n=4-6) were injected with PBS, J774 macrophages, or BMDMs (1x10^6^ cells/mouse) that were pre-incubated with or without 25 μg/mL acLDL for 24 hours to load cells with cholesterol (“CL-J774” or “CL-BMDM”). Peritoneal exudate cells here harvested by peritoneal lavage 24 or 48 hours post-injection and cell suspensions were prepared independently for flow cytometry analysis. (A) Representative gating strategy used to identify donor-derived macrophages. (B-C) Frequency of H-2Dd^+^ and CD45.1^+^ cells within perionteal exudate cells at 24 and 48 hours post-injection.

**Supplemental Figure 2. PEC Population Gating Strategies**

Wild type mice (n=9-13) were injected with PBS, J774 macrophages or BMDMs (1x10^6^ cells/mouse) that were pre-incubated with or without 25 μg/mL acLDL for 24 and 48 hours to load cells with cholesterol (“CL-J774” or “CL-BMDM”). Peritoneal exudate cells here harvested by peritoneal lavage 24 hours post-injection and cell suspensions were prepared independently for flow cytometry analysis. (**A**) F4/80^+^, (**B**) CD19^+^B220^lo^, CD19^+^B220^hi^, and (**C**) CD4^+^, CD8^+^ cells were determined by gating strategies indicated. **(D)** Total PEC numbers were counted in each cell suspension. Flow cytometry analysis of host peritoneal immune cell populations 24 and 48 hours following intraperitoneal injection. Donor macrophages were excluded from the F4/80^+^ macrophages gate based on H2D-d or CD45.1 expression. Frequencies of host F4/80^+^ macrophages, B-1 B cells, B-2 B cells, CD4^+^ T cells, and CD8^+^ T cells are shown as a percentatge of singlets. Statistical analyses were performed using two-way ANOVA followed by Tukey’s multiple comparisons test. * P < 0.05, **P<0.01.

**Supplemental Table 1. qPCR primer sequences**

| **Gene** | **Forward Primer (5’ – 3’)** | **Reverse Primer (5’ – 3’)** |
| --- | --- | --- |
| *Actb* | tcctagcaccatgaagatcaag | gactcatcgtactcctgcttg |
| *Ifng* | tcttggatatctggaggaactgg | tcaatgacgcttatgttgttgc |
| *Cd3e* | agtaatgagctggctgcgtc | caggatgccccagaaagtgt |
